# Supplementary material for: Reconciling Mining with the Conservation of Cave Biodiversity: A Quantitative Baseline to Help Establish Conservation Priorities
Source: PLoS One. 2016 Dec 20;11(12):e0168348. doi: 10.1371/journal.pone.0168348 (PMC5173368; doi:10.1371/journal.pone.0168348)
Supplement: S1 Dataset — (ZIP) [file pone.0168348.s002.zip › Taxa/Serra Norte/SN_2013/N4WS/Lista N4WS-04.pdf]

CAVIDADE N4WS-0004

Classe	Ordem	Fam/Outros	Gên/Outros	Espécie	S-U	U-S	Zon.
Arachnida	Acari	(Parasitengonina)		sp.7	X		P
Arachnida	Acari	Bdellidae		sp.1		X	E
Arachnida	Acari	Cosmochtoniidae		sp.2	X		E
Arachnida	Acari	Ixodidae	*Amblyomma*	sp.1	X		E
Arachnida	Acari	Laelapidae	*Cosmolaelaps*	sp.1		X	A
Arachnida	Acari	Mesostigmata		jovem		X	E
Arachnida	Amblypygi	Charinidae	*Charinus*	jovem	1		P
Arachnida	Amblypygi	Phrynidae	*Heterophrynus*	*longicornis*	6		E/P/A
Arachnida	Araneae	Araneidae		jovem	X		P
Arachnida	Araneae	Caponiidae		jovem	X	X	P
Arachnida	Araneae	Ochyroceratidae		jovem	X		E/P
Arachnida	Araneae	Oonopidae		jovem		X	E
Arachnida	Araneae	Pholcidae	*Mesabolivar*	*eberhard*		X	E
Arachnida	Araneae	Pisauridae		jovem	5		E
Arachnida	Araneae	Scytodidae	*Scytodes*	jovem	1	1	E/P
Arachnida	Araneae	Tetragnathidae		jovem		X	E
Arachnida	Araneae	Theridiidae	*Achaearanea*	sp.1	X		E/P
Arachnida	Araneae	Theridiosomatidae	*Ogulnius*	sp.1	X		E
Arachnida	Araneae	Theridiosomatidae	*Plato*	sp.1	X	X	E/P/A
Arachnida	Araneae	Theridiosomatidae		sp.1	X	X	E
Arachnida	Opiliones	Escadabiidae		sp.1	X	X	P/A
Arachnida	Opiliones	Sclerosomatidae	*Prionostema*	sp.1	X	X	E
Arachnida	Opiliones	Stygnidae	*Protimesius*	*gracilis*	2		E/P
Arachnida	Palpigradi	Eukoeneniidae	*Allokoenenia*	sp.1	X		P/A
Arachnida	Pseudoscorpiones	Chernetidae	*Spelaeochernes*	sp.1	X	X	E/P/A
Arachnida	Pseudoscorpiones	Chthoniidae	*Pseudochthonius*	sp.1	X		P
Arachnida	Pseudoscorpiones	Chthoniidae	*Pseudochthonius*	sp.3	X		P
Arachnida	Schizomida	Hubbardiidae	aff. *Rowlandius*	sp.1	X		P
Chilopoda	Geophilomorpha	Ballophilidae	*Ityphilus*	sp.2		1	A
Chilopoda	Scolopendromorpha	Cryptopidae	*Cryptops*	sp.1	1		E
Chilopoda	Scolopendromorpha	Scolopocryptopidae	*Newportia*	sp.1	1		E
Diplopoda	Glomeridesmida	Glomeridesmidae		sp.1	3		A
Diplopoda	Polydesmida	Pyrgodesmidae		sp.2	X	X	A
Diplopoda	Spirostreptida	Pseudonannolenidae	*Pseudonannolene*	sp.2	4	3	P
Entognatha	Collembola	Cyphoderidae		sp.1	X		P
Entognatha	Collembola	Entomobryidae		sp.6		X	E
Entognatha	Collembola	Isotomidae		sp.1	X		E/P
Entognatha	Collembola	Paronellidae		sp.1	X		E
Entognatha	Collembola	Paronellidae		sp.4	X	X	P/A
Entognatha	Collembola	Paronellidae		sp.6	X		E
Entognatha	Diplura	Campodeidae		sp.1	X		E
Insecta	Archaeognatha	Meinertellidae		sp.1		X	E
Insecta	Blattodea			jovem	6		E/P/A
Insecta	Coleoptera	Carabidae		jovem	X		E/P
Insecta	Coleoptera	Dytiscidae		sp.3	X		E
Insecta	Coleoptera	Dytiscidae		sp.19	X		E
Insecta	Coleoptera	Leiodidae		sp.5	X		E
Insecta	Coleoptera	Scydmaenidae		sp.2	X		E
Insecta	Coleoptera	Tenebrionidae		jovem	X		E/A

Insecta	Dermaptera	Pygidicranidae		sp.1		X	E
Insecta	Diptera	Cecidomyiidae	Lestreminae	sp.	X		E
Insecta	Diptera	Ceratopogonidae		jovem	X	X	E/P/A
Insecta	Diptera	Conopidae	*Stylogaster*	sp.	X		P
Insecta	Diptera	Culicidae	*Culex*	sp.		X	E
Insecta	Diptera	Muscidae		jovem	X		A
Insecta	Diptera	Mycetophilidae	*Mycomya*	sp.		X	A
Insecta	Diptera	Phoridae	Metopininae	sp.	X		E/P
Insecta	Diptera	Psychodidae	*Sciopemyia*	*sordellii*	X		E/P
Insecta	Diptera	Tipulidae	Tipulinae	sp.	X	X	E/P
Insecta	Heteroptera	Ceratocombidae		sp.1		X	E
Insecta	Heteroptera	Notonectidae		jovem	X		E
Insecta	Heteroptera	Reduviidae	Reduviinae	jovem	4	3	E
Insecta	Heteroptera	Schizopteridae	Schizopterinae	sp.1	X	X	A
Insecta	Heteroptera	Veliidae	*Paravelia*	sp.1	X		E/P
Insecta	Hymenoptera	Formicidae	*Apterostigma*	sp.1	X		E
Insecta	Hymenoptera	Formicidae	*Camponotus*	sp.1	X		P
Insecta	Hymenoptera	Formicidae	*Crematogaster*	sp.1	X		E/P
Insecta	Hymenoptera	Formicidae	*Crematogaster*	sp.3		X	E/P
Insecta	Hymenoptera	Formicidae	*Cyphomyrmex*	sp.2		X	P
Insecta	Hymenoptera	Formicidae	*Nylanderia*	sp.1	X		P
Insecta	Hymenoptera	Formicidae	*Nylanderia*	sp.3		X	E
Insecta	Hymenoptera	Formicidae	*Odontomachus*	sp.1	X		E
Insecta	Hymenoptera	Formicidae	*Pheidole*	sp.2	X		E/P
Insecta	Hymenoptera	Formicidae	*Solenopsis*	sp.1	X		P
Insecta	Hymenoptera	Formicidae	*Solenopsis*	sp.2		X	A
Insecta	Isoptera	Termitidae	*Nasutitermes*	sp.	X	X	P
Insecta	Lepidoptera	Noctuoidea		sp.12	1		E/P
Insecta	Lepidoptera			jovem		X	P
Insecta	Orthoptera	Phalangopsidae	*Paraclodes*	sp.	1		E
Insecta	Orthoptera	Phalangopsidae	*Phalangopsis*	sp.	39	57	A
Insecta	Psocoptera	Lepidopsocidae		jovem		X	E
Insecta	Psocoptera	Psocomorpha		jovem		X	E
Insecta	Psocoptera	Psyllipsocidae		jovem	X	X	E/P
Insecta	Thysanura	Nicoletiidae		sp.1	X	X	P
Gastropoda	Pulmonata	Bulimulidae	*Naesiotus*	sp.		X	A
Gastropoda	Pulmonata	Systrophiidae	*Happia*	sp.	X		A
Malacostraca	Isopoda	Armadillidae		sp.1		X	P
Malacostraca	Isopoda	Dubioniscidae		sp.1		X	E
Malacostraca	Isopoda	Philosciidae		sp.2	X		E
Malacostraca	Isopoda	Scleropactidae	*Circoniscus*	*buckupi*	X		P
Symphyla		Scutigerellidae	*Hanseniella*	sp.1	X		P
Amphibia	Anura	Strabomantidae	*Pristimantis*	cf. *fenestratus*	4		E/P
Mammalia	Chiroptera	Phyllostomidae	Glossophaginae	sp.	5		P
Mammalia	Chiroptera	Phyllostomidae		sp.		5	
Mammalia	Rodentia	Cricetidae	*Rhipidomys*	sp.		1	P
